# Extracellular Water Ratio and Phase Angle as Predictors of Exacerbation in Chronic Obstructive Pulmonary Disease

**DOI:** 10.3390/arm92030023

**Published:** 2024-05-31

**Authors:** An-Ni Xie, Wen-Jian Huang, Chih-Yuan Ko

**Affiliations:** 1 Department of Clinical Nutrition, Jinjiang Hospital of Traditional Chinese Medicine, Jinjiang 362200, China; 249612132@qq.com; 2 Department of Clinical Nutrition, The Second Affiliated Hospital of Fujian Medical University, Quanzhou 362000, China; 1074636286@qq.com; 3 Huidong Center for Chronic Disease Control, Huizhou 516300, China

**Keywords:** acute exacerbation of chronic obstructive pulmonary disease (AECOPD), phase angles, extracellular water ratios, exacerbation frequency, nutrition

## Abstract

**Highlights:**

**What are the main findings?**

- Significant differences in body composition parameters, including the extracellular water/total body water ratio (ECW/TBW) and phase angle (PhA), were observed between frequent and infrequent exacerbators.
- Increased exacerbation frequencies in COPD patients correlate with higher extracellular water ratios and lower phase angles.

**What is the implication of the main finding?**

- Body composition parameters such as ECW/TBW and PhA might serve as predictive markers for exacerbation risks in COPD patients, aiding targeted clinical interventions. However, larger studies are needed to confirm these findings and enhance their clinical relevance.
- Understanding these associations may enhance the management strategies for COPD, with the aim of reducing exacerbation frequencies and improving patient outcomes through tailored nutritional and therapeutic approaches.

**Abstract:**

Background: Chronic obstructive pulmonary disease (COPD), characterized by high-energy metabolism, often leads to malnutrition and is linked to exacerbations. This study investigates the association of malnutrition-related body composition and handgrip strength changes with exacerbation frequencies in COPD patients. Methods: We analyzed 77 acute exacerbation COPD (AECOPD) patients and 82 stable COPD patients, categorized as frequent and infrequent exacerbators. Assessments included body composition, handgrip strength, nutritional risk, dyspnea scale, and COPD assessment. Results: Among AECOPD patients, there were 22 infrequent and 55 frequent exacerbators. Infrequent exacerbators showed better muscle parameters, extracellular water ratio, phase angle, and handgrip strength. Significant differences in intracellular water, total cellular water, protein, and body cell mass were observed between groups. Logistic regression indicated that extracellular water ratio (OR = 1.086) and phase angle (OR = 0.396) were independently associated with exacerbation risk. Thresholds for exacerbation risk were identified as 0.393 for extracellular water ratio and 4.85° for phase angle. In stable COPD, 13 frequent and 69 infrequent exacerbators were compared, showing no significant differences in weight, muscle, and adipose parameters, but significant differences in extracellular water ratio, phase angle, and handgrip strength. Conclusions: These findings suggest that increased exacerbations in COPD patients correlate with higher extracellular water ratios and lower phase angles.

## 1. Introduction

Chronic obstructive pulmonary disease (COPD) involves persistent and incompletely reversible airflow obstruction, accompanied by respiratory symptoms [1]. COPD prevalence overall in 2019 was 10.3% [2]. In COPD, acute exacerbations are characterized by the acute deterioration of respiratory symptoms [1]. Patients with COPD still periodically experience exacerbations despite optimal therapy [3]. Exacerbations of COPD are associated with lung function decline, subsequent exacerbations, and high mortality [4,5,6]. Hence, reducing the frequency of COPD exacerbations is a key objective of COPD management.

As a result of COPD, individuals have impaired senses of taste and smell and impaired gastroenteric function, resulting in reduced calorie intake and malnutrition [7]. Malnutrition causes abnormal body composition, manifested as peripheral skeletal muscle atrophy [8]. There are substantial differences between patients with COPD and controls in terms of body composition [9,10,11]. Body composition analyzers (e.g., bioelectrical impedance analysis, BIA) are recommended by the European Respiratory Society’s Nutrition Working Group for patients with COPD [12]. Despite BIA having been used to investigate the body composition of COPD patients, little is known about the relationship between body composition and COPD exacerbations.

Additionally, chronic low-grade inflammation and negative energy balance can contribute to muscle atrophy, affecting muscle function in COPD patients [13]. Muscle dysfunction increases the risk of hospitalization and lowers health-related quality of life [14]. A clinical nutritional assessment includes handgrip strength (HGS) as a measure of muscle function [15]. COPD exacerbations were seen more frequently in patients with handgrip strength weakness than in those without [16]. Assessing HGS can help identify patients at risk of exacerbation [17]. In spite of this, it is still unclear whether HGS and body composition differ among patients with COPD stratified into different exacerbation phenotypes.

Thus, our study aimed to identify patients with higher exacerbation risk by comparing body composition and HGS between frequent exacerbators (FEs) (with ≥2 moderate exacerbations or at least one hospitalization for severe exacerbations in the previous year) and infrequent exacerbators (IEs) (with <2 moderate exacerbations without hospitalization for severe exacerbations in the prior year) [18]. We also aimed to explore the associations between these factors and COPD exacerbations.

## 2. Materials and Methods

### 2.1. Subjects and Study Protocol

From February 2021 to December 2022, a cross-sectional study was conducted on inpatients with AECOPD and outpatients with stable COPD in the Department of Respiratory and Critical Care Medicine, the Second Affiliated Hospital of Fujian Medical University. The Research Ethics Committee of the Second Affiliated Hospital of Fujian Medical University approved this study (ethics review number 2021-31), and the study participants provided written informed consent.

As inclusion criteria, patients had to be at least 45 years of age, men, and have previously been diagnosed with COPD according to the fixed ratio of the Global Initiative for Chronic Obstructive Lung Disease guidelines (GOLD; forced expiratory volume in 1s/forced vital capacity <0.7) [2]. Additional inclusion criteria for AECOPD patients included exacerbation meeting GOLD guidelines (a sudden acute worsening of respiratory symptoms beyond the daily state that requires adjustment of the treatment regimen) [2]. In this study, we excluded patients with contraindications to BIA (e.g., limb amputations), asthma, obstructive sleep apnea, active pulmonary tuberculosis, malignant tumors, serious renal or hepatic disorders, or systemic inflammatory or metabolic diseases.

We collected demographic data (e.g., age, education, and smoking status) and medical history data (e.g., comorbidities and frequency of exacerbations) from patients. We assessed dyspnea severity using the modified British Medical Research Council (mMRC) dyspnea scale, and evaluated health-related quality of life using the self-assessment test (CAT) [19,20]. We used nutritional risk screening 2002 (NRS-2002) to identify malnutrition risk [21,22].

### 2.2. Anthropometric Measurements

Body weight and height were measured with a digital electronic scale (Benbo AM-D1, Yongkang Time Electronic Technology Co. Ltd., Zhejiang, China) and stadiometer (HT-01, Hengqi Group Co. Ltd., Guangdong, China), respectively.

HGS was measured using a spring dynamometer (EH101, Hengqi Group Co., Ltd., Guangdong, China). The patients were instructed to remain in the sitting position with their forearms neutrally positioned, their elbows bent at 90 degrees, and to squeeze the dynamometer with their dominant hand as hard as they could. For each patient, we measured HGS twice, approximately one minute apart, and calculated the average of both measurements.

We measured body composition using a portable multifrequency BIA (InbodyS10, Biospace, Seoul, Republic of Korea). We conducted BIA to determine body composition parameters, including intracellular water (ICW), extracellular water (ECW), total body water (TBW), protein, mineral, bone mineral content (BMC), body cell mass (BCM), arm circumference (AC), arm muscle circumference (AMC), soft lean mass (SLM), skeletal muscle mass (SMM), skeletal muscle mass index (SMI), fat-free mass (FFM), fat mass (FM), percent body fat (PBF), visceral fat area (VFA), waist circumference, extracellular water ratio (ECW/TBW), and phase angle (PhA). We calculated the fat mass index (FMI) or fat-free mass index (FFMI) by dividing FM or FFM by height squared [23]. The tests were conducted under standardized conditions (bare feet, minimal clothing, empty bladder, and fasting). Our study was consistent with a previous study in terms of the measurement method [24].

### 2.3. Statistical Analysis

We conducted all analyses using Statistical Package for Social Sciences (SPSS, version 26.0) and considered a *p*-value of less than 0.05 statistically significant. The descriptive results were expressed as mean ± standard deviation (SD) or median [interquartile range (IQR)] for continuous variables, and frequency (percentage) for categorical variables. We used Student’s *t*-test or the Mann–Whitney U test to compare continuous variables, and a chi-square test to compare categorical variables.

We performed binary logistic regression analysis with selected parameters through collinearity analysis. In the regression models, odds ratios (ORs) with 95% confidence intervals (CIs) were used to estimate the risk of COPD exacerbations associated with body composition and HGS. The receiver operating characteristic (ROC) curves for body composition were analyzed to estimate the threshold for COPD exacerbations.

## 3. Results

### 3.1. Characteristics of Participants

**Figure 1:**
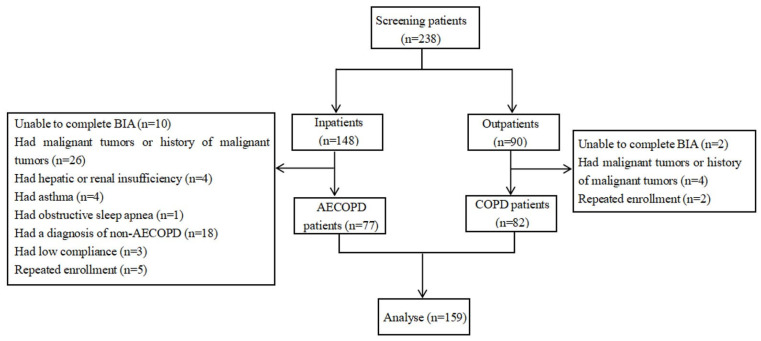
Flow diagram of study subject inclusion. BIA: bioelectrical impedance analysis.

In this study, we initially recruited 148 inpatients and 90 outpatients. However, the final cohort was adjusted due to several exclusions. Specifically, 12 subjects were unable to complete BIA due to instrumental limitations, 30 had a malignant tumor or a history of such, 4 were suffering from hepatic or renal insufficiency, 4 had asthma, 1 was diagnosed with obstructive sleep apnea, 18 in the inpatient group were diagnosed with non-AECOPD, 3 displayed low compliance, and 7 were repeat enrollees. Consequently, the final study group comprised 159 subjects, including 77 with AECOPD and 82 with stable COPD. The enrollment process is detailed in Figure 1. Significant differences were observed between COPD and AECOPD patients in several areas, including age, weight, body mass index (BMI), current smoker ratio, disease duration, NRS-2002, mMRC, and CAT (*p* < 0.05; Table 1).

### 3.2. Body Composition and HGS in AECOPD Patients with Different COPD-Exacerbating Frequencies

Table 2 shows body composition and HGS differences in AECOPD patients based on COPD exacerbation frequency. Significant variances were found in total body water, intracellular water, protein, body cell mass, the ECW/TBW ratio, and PhA between groups (*p* < 0.05). Muscle parameters (i.e., soft lean mass, skeletal muscle mass, skeletal muscle mass index, fat-free mass, and fat-free mass index) and HGS were better in the IE group compared to the FE group (*p* < 0.05).

### 3.3. Factors Associated with COPD-Exacerbating Frequency in AECOPD Patients

**Figure 2:**
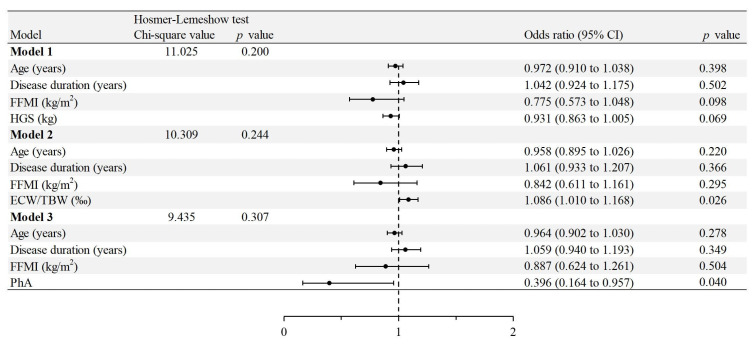
Factors associated with chronic obstructive pulmonary disease (COPD)-exacerbating frequency of patients with acute exacerbations of COPD in binary logistic regression models. FFMI: fat-free mass index; HGS: handgrip strength; ECW/TBW: extracellular water/total body water; PhA: phase angle.

In this study, three regression models were developed using binary logistic regression analysis, following collinearity analysis of selected parameters, to identify factors influencing COPD exacerbation frequency. Adjusting for covariates revealed that the ECW/TBW ratio (OR = 1.086; 95% CI: 1.010, 1.168; *p* = 0.026) positively influenced COPD exacerbation, whereas PhA (OR = 0.396; 95% CI: 0.164, 0.957; *p* = 0.040) had a negative impact (Figure 2).

### 3.4. ROC Curve Analyses and Optimum Critical Values in AECOPD Patients

**Figure 3:**
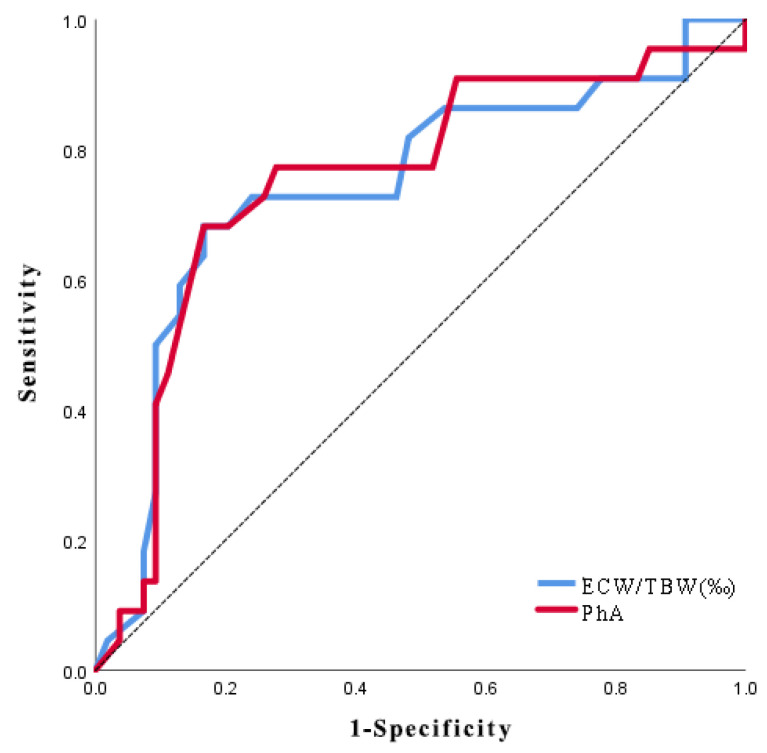
Receiver operating characteristic curves for extracellular water ratio and phase angle in identifying exacerbation risks in patients with acute exacerbation of chronic obstructive pulmonary disease. ECW/TBW: extracellular water/total body water; PhA: phase angle.

ROC curve analyses determined the thresholds for the ECW/TBW ratio and PhA associated with COPD exacerbations. The areas under the ROC curves (AUC) were 0.744 (95% CI, 0.611 to 0.876) for ECW/TBW and 0.753 (95% CI, 0.624 to 0.882) for PhA. At a threshold of 0.393 for ECW/TBW, sensitivity was 68.2% and specificity was 83.3% in identifying COPD-exacerbating phenotypes. Similarly, for PhA at a threshold of 4.85°, sensitivity and specificity were 68.2% and 83.3%, respectively (Figure 3).

### 3.5. Body Composition and HGS in COPD Patients with Different COPD-Exacerbating Frequencies

Due to the severe illness of COPD patients in the FE group, regular outpatient visits were challenging, resulting in only 13 FE group patients being included in the analysis. Statistical analysis comparing body composition and HGS between FE and IE groups showed no significant differences in weight, muscle, and adipose parameters (*p* ≥ 0.05). However, differences in the ECW/TBW ratio (*p* = 0.003), PhA (*p* = 0.002), and HGS (*p* = 0.008) were statistically significant (Table 3).

## 4. Discussion

The relationship between body composition and COPD exacerbations has been extensively researched [14,25,26]. However, little is known about the relationship between PhA and/or ECW/TBW and COPD exacerbations. Our study examined the differences in body composition and HGS between patients with different COPD-exacerbating frequencies. This study demonstrated that patients who experience fewer COPD exacerbations tend to have a better PhA and a more favorable ECW/TBW ratio.

The study included AECOPD patients with more severe conditions, who were older and had longer disease duration compared to the stable COPD population. Additionally, AECOPD patients had poorer nutritional status and more significant dyspnea symptoms. Patients with AECOPD were less likely to be current smokers. A worsening health condition may motivate patients to quit smoking. According to a previous study, patients with mild COPD had a lower likelihood of quitting smoking [27].

Patients with COPD were monitored nutritionally using the BIA device in clinical practice. Analyzing body composition with BIA is quick, simple, noninvasive, and relatively inexpensive, and it can be used to detect malnutrition and the effectiveness of nutrition interventions [28]. In this study, we partially examined the associations between BIA-derived body composition parameters and exacerbation frequency. Our findings indicated differences in water, protein, body cell mass, muscle parameters, ECW/TBW, and PhA of AECOPD patients stratified into different exacerbation phenotypes. Studies have shown that COPD exacerbations are associated with a decline in lean-related indicators [14,25]. However, based on binary logistic regression analyses, FFMI did not appear to be associated with COPD exacerbations. We speculated that FFMI is explained by its dependence on prediction equations and BIA measurements which assume a balanced fluid distribution [28].

The progression of alveolar hypoxia in patients with COPD leads to chronic hypoxic pulmonary vasoconstriction, remodeling of the pulmonary vascular system, and subsequent pulmonary hypertension. Increasing the right ventricle load in response to pulmonary vascular resistance leads to right heart hypertrophy. Adaptive hypertrophy of the heart causes cardiac dysfunction and peripheral edema [29,30]. In hospitalized patients with COPD, biomarkers of cardiac dysfunction such as N-terminal pro-brain natriuretic peptide (NT-proBNP), and troponin T are frequently elevated. Patients with abnormal NT-proBNP and troponin T levels had a 15-fold higher 30-day mortality compared with those with normal values [31]. One of the manifestations of COPD is edema, in which COPD patients are hyperhydrated despite post-fluid volume control [32]. A measurement of ECW/TBW using BIA can indicate hydration status [33]. TBW refers to the overall volume of water contained within an individual’s body, comprising approximately 60% of an adult’s body weight. It is a fundamental parameter in body composition analysis, divided into ICW and ECW compartments. Accurate TBW measurements are essential for assessing hydration status, nutritional status, and overall health, particularly in clinical settings where precise fluid balance evaluation is critical [34]. Understanding and measuring TBW and its components (ICW and ECW) are crucial in both clinical and research contexts [35,36]. COPD patients with higher ECW/TBW had worse clinical outcomes [37]. Based on our finding that 0.393 was a critical value for ECW/TBW, the BIA tool might be useful for identifying patients at risk of COPD exacerbations.

In this study, a cut-off value of 4.85° for the PhA was identified as indicative of patients with frequent exacerbations, thereby enabling more targeted clinical interventions. PhA is derived from electrophysiological data and is not dependent on prediction equations from the BIA machine [28]. A healthy cell membrane acts as a good capacitor, with PhA reflecting the cell membrane’s ability to store and delay current flow. Low PhA indicates cell death or compromised membrane permeability, which can negatively impact clinical outcomes. Thus, PhA serves as a marker of cellular health and nutritional status [38]. Conditions like sarcopenia and malnutrition are associated with reduced PhA, highlighting its role in indicating the prevalence of these conditions [28,39].

Exacerbation of COPD is a serious cause of readmission and mortality in patients, and it substantially affects quality of life [1]. Individuals with COPD who have low PhA and/or high ECW/TBW have been shown to have a worse outcome, with frequent acute exacerbations. Our findings indicated that BIA might be used to identify COPD patients with specific nutritional phenotypes and their risk of future acute exacerbations. As a result, the risk of COPD exacerbation could be controlled by timely treatment, reducing the rate of disability due to frequent exacerbations and achieving tertiary prevention. In a review of research, oral nutritional supplementation (ONS) benefits body weight and FFMI [7,40], but we are unsure whether dietary interventions or ONS improve ECW/TBW or PhA in COPD patients. These findings suggest that interventional studies are needed to investigate the role of changes in body composition in reducing COPD exacerbations.

This study faced several limitations. Firstly, the sample size was relatively small, especially among stable COPD patients experiencing frequent exacerbations. Conducting a follow-up study with a larger sample size could confirm potential markers for COPD exacerbation risk and produce more robust results. Secondly, all patients included were previously diagnosed, meaning no repeat pulmonary function tests or blood biochemical analyses were conducted. Additionally, the data were cross-sectional, preventing the establishment of causality. Lastly, the absence of female participants is noteworthy, likely due to the lower prevalence of COPD among women in Quanzhou, possibly linked to occupational or cigarette exposure. Future research should prioritize including female COPD patients, considering their distinct body composition from males, necessitating further investigation.

## 5. Conclusions

The present study indicates that the ECW/TBW ratio and PhA could be key in identifying patients at a higher risk of COPD exacerbations, facilitating targeted clinical interventions. These insights have potential implications for future COPD management strategies.

## Figures and Tables

**Table 1:** Baseline characteristics of the participants.

Variables	AECOPD Participants (n = 77)	COPD Participants (n = 82)	*p* Value
Age (years)	67.0 ± 8.8	62.9 ± 8.3	0.002
Weight (kg)	51.6 ± 10.7	58.8 ± 12.4	<0.001
BMI (kg/m^2^)	18.6 ± 3.4	21.4 ± 4.1	<0.001
Education			0.613
Pre-school or primary	44 (57.1%)	41 (50.0%)	
Secondary	32 (41.6%)	39 (47.6%)	
Higher	1 (1.3%)	2 (2.4%)	
Occupational exposure	49 (63.6%)	47 (57.3%)	0.416
Smoke exposure			
Current smoker	12 (15.6%)	27 (32.9%)	0.011
Tobacco consumption (package/year)	57.5 [43.4, 88.5]	48.8 [36.8, 74.3]	0.096
Alcohol exposure	30 (39.0%)	35 (42.7%)	0.633
Disease duration (years)	5.5 [3.0, 10.0]	4.0 [1.4, 6.0]	<0.001
NRS-2002			
<3 points	21 (27.3%)	59 (72.0%)	<0.001
≥3 points	56 (72.7%)	23 (28.0%)	
mMRC			<0.001
Grade 0	2 (2.6%)	19 (23.2%)	
Grade 1	7 (9.1%)	14 (17.1%)	
Grade 2	8 (10.4%)	27 (32.9%)	
Grade 3	35 (45.5%)	17 (20.7%)	
Grade 4	25 (32.5%)	5 (6.1%)	
CAT	21.0 [16.0, 26.0]	13.5 [9.0, 18.0]	<0.001

BMI: body mass index; NRS-2002: nutritional risk screening 2002; mMRC: the modified British Medical Research Council dyspnea scale; CAT: chronic obstructive pulmonary disease assessment test.

**Table 2:** Body composition and handgrip strength in patients with acute exacerbation of chronic obstructive pulmonary disease according to exacerbating frequencies.

Variables	Infrequent Exacerbators (n = 22)	Frequent Exacerbators (n = 55)	*p* Value
Height (cm)	166.59 ± 5.60	166.24 ± 6.01	0.815
Weight (kg)	53.61 ± 10.42	50.82 ± 10.91	0.309
BMI (kg/m^2^)	19.25 ± 3.22	18.34 ± 3.52	0.304
ICW (kg)	19.46 ± 3.25	17.73 ± 2.71	0.020
ECW (kg)	12.56 ± 1.92	11.84 ± 1.74	0.114
TBW (kg)	32.02 ± 5.14	29.57 ± 4.41	0.039
Protein (kg)	8.40 ± 1.40	7.67 ± 1.18	0.024
Mineral (kg)	2.90 ± 0.45	2.75 ± 0.39	0.128
BMC (kg)	2.39 ± 0.36	2.27 ± 0.31	0.137
BCM (kg)	27.86 ± 4.65	25.39 ± 3.89	0.021
AC (cm)	26.10 [24.40, 28.35]	25.55 [22.65, 27.38]	0.313
AMC (cm)	24.10 [22.68, 26.38]	23.10 [21.45, 24.50]	0.160
SLM (kg)	40.93 ± 6.62	37.71 ± 5.67	0.036
SMM (kg)	23.38 ± 4.23	21.12 ± 3.54	0.020
SMI (kg/m^2^)	6.43 ± 1.02	5.83 ± 0.86	0.011
FFM (kg)	43.33 ± 6.95	39.97 ± 5.94	0.037
FFMI (kg/m^2^)	15.56 ± 2.02	14.44 ± 1.78	0.019
FM (kg)	10.28 ± 5.26	10.85 ± 6.64	0.723
FMI (kg/m^2^)	3.69 ± 1.82	3.90 ± 2.33	0.699
PBF (%)	17.75 [13.90, 23.52]	18.40 [14.15, 25.15]	0.606
VFA (cm^2^)	46.75 [38.05, 69.58]	50.85 [37.40, 75.85]	0.354
Waist circumference (cm)	74.45 [68.78, 78.90]	72.55 [69.98, 82.48]	0.918
ECW/TBW	0.390 [0.387, 0.400]	0.402 [0.396, 0.405]	0.001
PhA	4.90 [4.45, 5.20]	4.00 [3.48, 4.55]	0.001
HGS (kg)	24.64 ± 8.72	19.39 ± 7.03	0.008

BMI: body mass index; ICW: intracellular water; ECW: extracellular water; TBW: total body water; BMC: bone mineral content; BCM: body cell mass; AC: arm circumference; AMC: arm muscle circumference; SLM: soft lean mass; SMM: skeletal muscle mass; SMI: skeletal muscle mass index; FFM: fat-free mass; FFMI: fat-free mass index; FM: fat mass; FMI: fat mass index; PBF: percent body fat; VFA: visceral fat area; ECW/TBW: extracellular water/total body water; PhA: phase angle; HGS: handgrip strength.

**Table 3:** Body composition and handgrip strength in patients with chronic obstructive pulmonary disease according to exacerbating frequencies.

Variables	Infrequent Exacerbators (n = 69)	Frequent Exacerbators (n = 13)	*p* Value
Height (cm)	165.58 ± 4.92	165.08 ± 5.50	0.744
Weight (kg)	58.04 ± 9.71	60.51 ± 21.93	0.513
BMI (kg/m^2^)	21.12 ± 3.18	22.12 ± 7.60	0.433
ICW (kg)	20.97 ± 3.13	19.47 ± 3.33	0.122
ECW (kg)	12.85 ± 1.72	12.29 ± 2.10	0.307
TBW (kg)	33.82 ± 4.82	31.76 ± 5.41	0.172
Protein (kg)	9.07 ± 1.35	8.41 ± 1.45	0.115
Mineral (kg)	2.99 ± 0.45	2.86 ± 0.43	0.319
BMC (kg)	2.45 ± 0.40	2.35 ± 0.36	0.389
BCM (kg)	30.03 ± 4.49	27.89 ± 4.79	0.123
AC (cm)	28.10 [26.40, 30.40]	28.20 [25.10, 29.50]	0.566
AMC (cm)	25.40 [24.30, 26.70]	25.20 [22.75, 26.15]	0.419
SLM (kg)	43.42 ± 6.24	40.68 ± 6.92	0.158
SMM (kg)	25.35 ± 4.08	23.39 ± 4.34	0.120
SMI (kg/m^2^)	7.13 ± 1.84	6.54 ± 0.99	0.262
FFM (kg)	45.88 ± 6.51	43.04 ± 7.26	0.162
FFMI (kg/m^2^)	16.69 ± 1.92	15.74 ± 2.20	0.116
FM (kg)	12.16 ± 5.71	17.47 ± 17.82	0.308
FMI (kg/m^2^)	4.44 ± 2.10	6.38 ± 6.39	0.300
PBF (%)	20.00 [15.00, 24.70]	23.20 [15.20, 29.95]	0.303
VFA (cm^2^)	47.10 [31.90, 67.80]	67.20 [30.00, 83.10]	0.334
Waist circumference (cm)	76.40 [71.20, 83.80]	78.00 [68.20, 83.60]	0.979
ECW/TBW	0.380 [0.377, 0.385]	0.385 [0.382, 0.392]	0.003
PhA	5.90 [5.30, 6.30]	5.30 [4.65, 5.55]	0.002
HGS (kg)	32.38 ± 7.18	26.53 ± 6.07	0.008

Abbreviations are as in Table 2.

## Data Availability

The datasets generated and/or analyzed during the current study are not publicly available due to confidentiality requirements but are available from the corresponding author on reasonable request.
